# Systematic and synthetic biology insights into copper homeostasis in Escherichia coli

**DOI:** 10.1016/j.isci.2025.113715

**Published:** 2025-10-06

**Authors:** Zhiqiang Chen, Yu Fu, Jiajia Li, Jin Wang, Xiaona Fang

**Affiliations:** 1 School of Chemistry, Northeast Normal University, Changchun, Jilin 130024, P.R. China; 2 State Key Laboratory of Electroanalytical Chemistry, Changchun Institute of Applied Chemistry, Chinese Academy of Sciences, Changchun, Jilin 130022, P.R. China; 3 School of Applied Chemistry and Engineering, University of Science and Technology of China, Hefei, Anhui 230022, P.R. China; 4 Department of Chemistry and Physics, State University of New York at Stony Brook, Stony Brook, NY 11794-3400, USA

**Keywords:** Biological sciences, Medical Microbiology, Microbial metabolism, Microbial physiology, Molecular microbiology

## Abstract

Copper serves as an essential trace element in biological systems through its association with metalloenzymes. Two major regulatory systems (cueR and cus) participate in the transcriptional regulation of copper homeostasis genes, yet critical mechanisms remain poorly understood. We systematically investigated E. coli’s copper response using systems and synthetic biology approaches, revealing more detailed mechanisms. Our results suggested that the cus system specifically responds to Cu^+^ but not Cu^2+^, with Cu^2+^ to Cu^+^ reduction occurring primarily in the cytoplasm. Cu^+^ in the periplasmic space mainly originates from CopA-mediated export through the cytoplasm. We discovered that CusR exhibits signal crosstalk, causing baseline expression, which can be regulated by optimal CusS concentrations. CusS functions as both a kinase and a phosphatase depending on Cu^+^ presence. We quantified E. coli’s response to various extracellular Cu^2+^ concentrations, ultimately clarifying the relationship between the two copper ion response systems. These findings provide comprehensive insights into bacterial copper homeostasis mechanisms.

## Highlights

• CueO mediates copper detoxification primarily via enzymatic oxidation rather than chelation
• The Cus system is exclusively triggered by Cu^+^ and not Cu^2+^
• CusS displays kinase and phosphatase activities regulated by Cu^+^
• The Cue and Cus systems operate in a concentration-dependent synergistic manner

## Graphical abstract

**Figure:**
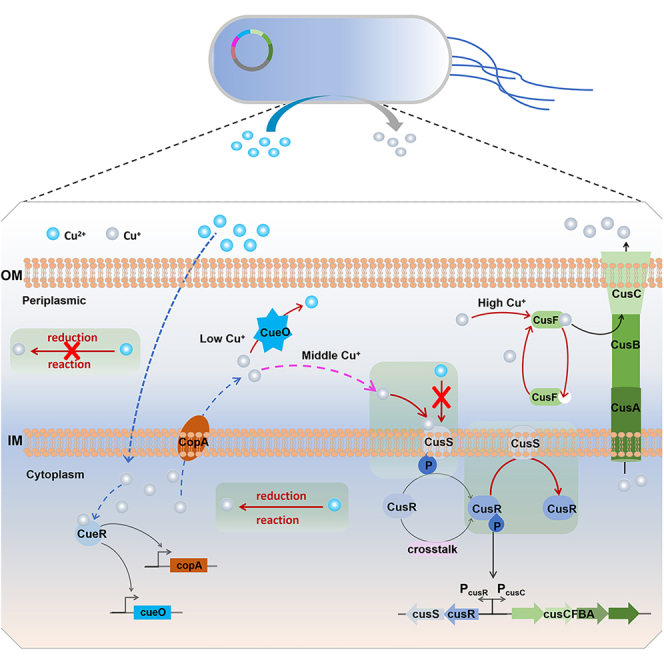

## Introduction

Copper is an essential trace element for living organisms, playing a crucial role in many biological processes, such as aerobic respiration.^1^^,^^2^^,^^3^^,^^4^^,^^5^ However, the dual nature of copper makes its homeostasis within bacteria particularly important: on one hand, a copper deficiency can cause dysfunctions in multiple enzymes, impacting growth and reproduction^2^^,^^6^; on the other hand, high concentrations of copper ions are highly cytotoxic, leading to oxidative stress and cell damage, and even causing bacterial death.^2^^,^^3^^,^^4^^,^^7^ Maintaining copper balance is vital for biological functions, prompting bacteria to develop sophisticated copper homeostasis mechanisms that tightly regulate copper ion intake and expulsion. These mechanisms ensure an adequate supply for copper protein biosynthesis and the removal of excess copper ions.^5^ The copper homeostasis system in bacteria is regulated by a complex network of proteins and genes. Understanding their functions can provide insights into the adaptive strategies employed by bacteria under various physiological conditions. Meanwhile, elucidating the copper regulation mechanism provides potential targets for developing novel antimicrobial drugs.^7^^,^^8^^,^^9^^,^^10^ Disrupting bacterial copper homeostasis could inhibit bacterial growth and survival. For instance, small molecule inhibitors targeting copper ion transport proteins or efflux pumps could serve as candidate antimicrobial agents.^2^^,^^4^^,^^7^^,^^8^^,^^9^^,^^10^ Therefore, elucidating the mechanisms of copper homeostasis is crucial for comprehending how bacteria regulate the balance of metal ions, which is fundamentally important in bacterial physiology and biochemistry. Moreover, it also holds significant potential for environmental applications.

*E. coli* is one of the most prevalent bacteria in the natural environment. The Cus and Cue systems in *E. coli* collaborate to regulate cellular resistance to copper ions and maintain intracellular copper ion homeostasis. The Cue system is based on CueR regulation, while the Cus system is based on the CusRS two-component system. These systems encompass several genes, including *cueO*, *copA*, *cusS*, *cusR*, and *cusCFBA*.^11^^,^^12^^,^^13^^,^^14^ As previously reported, the Cue system, with CueR as a transcription factor, activates the promoter of P_*copA*_ in response to copper ions, thereby regulating the expression of CopA and CueO.^15^^,^^16^ CueO is a multicopper oxidase that detoxifies copper by oxidizing Cu^+^ to less toxic Cu^2+^.^17^^,^^18^^,^^19^^,^^20^^,^^21^^,^^22^^,^^23^ CopA is a copper-transporting P-type ATPase that functions to export excess copper ions from the cytoplasm into the periplasmic space.^24^^,^^25^^,^^26^^,^^27^^,^^28^ In the Cus system, CusS binds to copper ions, undergoes self-phosphorylation, and subsequently transfers the phosphate group to CusR, resulting in the phosphorylation of CusR. The phosphorylated CusR (CusR-P) activates the promoters P_*cusC*_ and P_*cusR*_, thereby promoting the expression of CusS and CusR and establishing a positive feedback loop.^29^^,^^30^^,^^31^^,^^32^^,^^33^^,^^34^^,^^35^ Concurrently, it also enhances the expression of several proteins, namely CusCFBA, which function to expel copper ions from the cytoplasm and the periplasmic space to the exterior of the cell.^36^^,^^37^^,^^38^^,^^39^

Although the Cus system and Cue system in *E. coli* have been studied for a long time, certain key regulatory processes remain unclear. CueO has been reported to be involved in copper ion homeostasis regulation and protection against oxidative stress. The loss of the *cueO* gene hinders the oxidation of Cu^+^, making cells more sensitive to external copper and increasing the overall cellular accumulation of copper.^14^^,^^16^^,^^17^^,^^18^^,^^19^^,^^20^^,^^21^^,^^22^^,^^23^ Current research on CopA primarily concentrates on its structure, such as its dependence on the CopZ copper chaperone protein to transfer Cu^+^ to its transmembrane metal binding site. Additionally, CopA is capable of transferring Cu^+^ directly to CusF for excretion.^24^^,^^25^^,^^28^ Similarly, the majority of research on CusS also focuses on its structure. The metal binding sites in CusS are categorized into the interface binding site and the internal binding site. Notably, only the interface binding site is responsible for metal-induced CusS dimerization and is pivotal in initiating auto-phosphorylation. Furthermore, the H271 residues of CusS and the D51 residues of CusR are essential for autophosphorylation and the transfer of phosphate groups, and CusS undergoes a *cis*-autophosphorylation mechanism.^31^^,^^40^^,^^41^ Simultaneously, CopS in Pseudomonas aeruginosa, which is a homologous protein of CusS, has previously been reported to possess phosphatase activity.^42^ However, it is currently unclear whether CusS also possesses phosphatase activity. Regarding the copper efflux system, the focus is on the CusCFBA complex protein in *E. coli*. Most reports have studied its structure and transport route information.^36^^,^^37^ Based on the known structural information of CusCFBA proteins, the assembly, interactions, and the relationship between the structure and function of these proteins have been discussed in previous studies.^38^ Furthermore, according to the report, the absence of CusCBA does not enhance copper sensitivity, and while CueO can replace CusCBA, it cannot substitute for CopA.^43^ Copper efflux pumps and oxidative protein repair systems are co-regulated, with methionine being a crucial residue linked to the copper transport protein CusF.^39^

Previous research established that CusR transcriptional activity exhibits a dose-dependent response to increasing intracellular Cu^+^ concentrations.^35^^,^^40^^,^^44^^,^^45^^,^^46^^,^^47^ Outten et al.,^44^ provided the direct evidence that CusR activation is driven by periplasmic Cu^+^, demonstrating a marked increase in activity under strictly anaerobic conditions—where Cu^+^ predominates due to the absence of oxidative Cu^2+^ conversion. This finding was further substantiated by Ishihara et al.,^48^ who showed that CusR expression correlates directly with intracellular Cu^+^ bioavailability: transcriptional activity was diminished in *ΔcopA* mutants (impaired cytosolic Cu^+^ export) and hyperactivated in ΔcueO mutants (periplasmic Cu^+^ accumulation). Their systems-level modeling quantitatively established Cu^+^ as the dominant effector. Structural insights into this specificity were elucidated by Affandi et al.,^40^ who resolved the CusS periplasmic domain in complex with Cu^+^, identifying a conserved metal-binding pocket (His42-Phe43-His176) critical for Cu^+^ coordination. Mutagenesis of this site abolished copper resistance, confirming its functional necessity. Complementary genetic studies by Gudipaty et al.^45^ demonstrated that the CusS-dependent induction of the cusCFBA operon requires intact Cu^+^ sensing. These collective findings support the conclusion that CusS primarily recognizes periplasmic Cu^+^. while it is unknown whether CusS has no response at all to Cu^2+^. Once Cu^2+^ enters the cell, it is reduced to Cu^+^. However, the precise location of this reaction, whether it occurs in the periplasmic space or within the cytoplasm, remains ambiguous. It is also unclear whether CusS possesses phosphatase activity such as CopS. Furthermore, the interconnection and cooperation between the two copper detoxification pathways, CueO and CusCFBA, have not been fully elucidated. Recent studies have been focused on understanding protein function based on their structure, but they have not addressed these questions. In this work, we systematically investigated the response and regulatory processes of *E. coli* to copper ions through systems and synthetic biology approaches. We endeavor to elucidate the functions and roles of the aforementioned genes to complement and improve the Cue and Cus systems from a holistic perspective. Based on our experimental observations, CusS appears to specifically respond to Cu^+^, with no detectable response to Cu^2+^. Our analysis suggested that Cu^2+^ was primarily reduced in the cytoplasm, while Cu^+^ present in the periplasmic space was predominantly excreted by CopA from the cytoplasm. We also demonstrated that CusR exhibited signal crosstalk and may undergo phosphorylation by other proteins in the absence of copper ions. Additionally, we discovered that in addition to kinase activity, CusS also possesses phosphatase activity, enabling it to dephosphorylate CusR-P. Through gene knockout experiments, we inferred that, for the detoxification of Cu^+^ in the periplasmic space, CueO and CusCFBA worked cooperatively and could be substituted for each other at low concentrations of copper ions, while CusCFBA played a predominant role at high concentrations. Finally, by comparing the Cue and Cus systems, we hypothesized that the Cus system was activated only when the Cue system responded to low concentrations of copper ions. This work enhances our understanding of the mechanisms underlying copper ion tolerance in bacteria. Moreover, we anticipate that our research can contribute to the development of strategies for mitigating the deleterious effects of excessive copper ion exposure in the future.

## Results and discussion

### Dominant role of CueO oxidation, not chelation, in copper homeostasis confirms CusS specificity for Cu^+^

**Figure 1:**
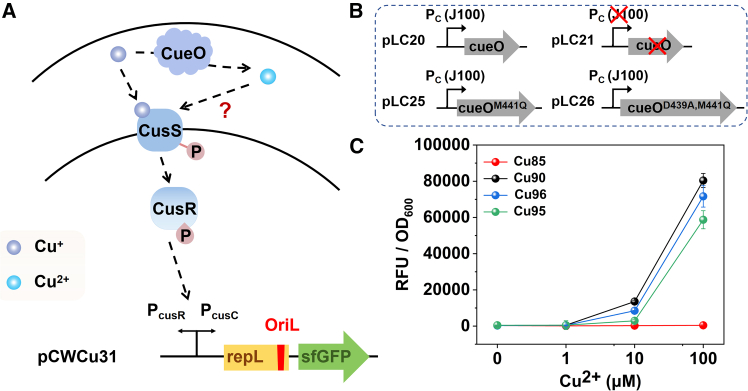
Sensing of CusS to copper ions (A) The schematic diagram of the response of CusS to Cu^+^/Cu^2+^ in the *cus* system. (B) The schematic diagram was the genetic elements of plasmids pLC20, pLC21, pLC25, and pLC26. (C) The response curves of strains Cu85 (*DH5α::ΔcusCFBA*/pCWCu31/pLC20), Cu90 (*DH5α::ΔcusCFBA*/pCWCu31/pLC21), Cu95 (*DH5α::ΔcusCFBA*/pCWCu31/pLC25) and Cu96 (*DH5α::ΔcusCFBA*/pCWCu31/pLC26). Measurements were performed with 3 replicates; error bars denote standard deviation.

The bacterial CusRS two-component system primarily regulates copper ion homeostasis in *E. coli*. The principal mechanism involves the binding of copper ions to the periplasmic sensing domain of CusS, which triggers a phosphorylation reaction in CusS itself.^35^ The phosphate group is then transferred to CusR (a response regulator), which activates the transcription of the cusCFBA and cusRS operons. This helps the cell adapt to high copper environments and maintain copper ion homeostasis.^30^ According to previous reports, the Cus system respond to Cu^+^ and regulate intracellular copper balance. However, the CusRS two-component system-based copper sensor can also detect Cu^2+^. This raises the question: does CusS only respond to Cu^+^, or can it also directly respond to Cu^2+^ (Figure 1A)? To detect the copper-sensing of CusS, we inserted sfGFP downstream of the promoter P_*cusC*_ as a reporter gene. Meanwhile, a signal amplifier gene, *repL*, was introduced to achieve a higher signal,^49^ generating the plasmid pCWCu31 (Figure 1A). The *cusCFBA* genes, known for expelling copper ions, were knocked out to retain copper ions in the periplasmic space and facilitate the response of CusS, creating the modified strain of *DH5*α*::ΔcusCFBA*. Plasmid pCWCu31 was then transformed into *DH5α::ΔcusCFBA*, resulting in the strain Cu39(*DH5*α*::ΔcusCFBA/*pCWCu31). To investigate the copper detoxification function of CueO, we constructed two isogenic plasmid variants (Figure 1B): pLC20 carrying the *cueO* gene under control of the strong constitutive promoter BBa_*J23100 (J100)*, and its corresponding control plasmid pLC21 generated by the precise deletion of both the *J23100* promoter and the entire *cueO* coding sequence. The pLC20 construct enables an overexpression of CueO, a multicopper oxidase that converts toxic Cu^+^ to less harmful Cu^2+^, while pLC21 serves as a rigorously controlled background reference. Ultimately, pLC20 and pLC21were individually introduced into Cu39 to establish Cu85 and Cu90, respectively.

We evaluated the responses of Cu85 and Cu90 to Cu^2+^ using the following procedure. The overnight bacterial cultures were diluted 1:100 in fresh LB medium (supplied with ampicillin and chloramphenicol) and incubated at 37°C until the optical density at 600 nm (OD_600_) reached approximately 0.6. The cultures were then exposed to varying concentrations of Cu^2+^ for 5 h prior to analysis (Unless otherwise specifically stated, all gene expression assays in this study were conducted under these standardized culture conditions). As the results illustrated in Figure 1C, the responses of Cu85 consistently failed to produce a detectable output signal, whereas Cu90 exhibited a significant signal response. This suggested that the excessive CueO in Cu85 oxidized most Cu^+^ in the periplasmic space to Cu^2+^, confirming that the CusS can only sense Cu^+^ but not Cu^2+^. Additionally, despite only Cu^2+^ being added to the culture, Cu90 still showed a strong output, confirming that Cu^2+^ can be reduced to Cu^+^ inside the cell and transported to the periplasmic space, where it was sensed by CusS, consistent with previous reports.^1^^,^^50^ However, this also revealed that in wild-type strains with normal CueO expression, even if CueO expression was upregulated due to CueR regulation, a significant amount of Cu^+^ still remained in the periplasmic space, suggesting a limited capacity of CueO for copper detoxification.

However, previous studies have demonstrated that CueO, as a multi-copper oxidase, not only catalyzes the oxidation of Cu^+^ to Cu^2+^, but also possesses copper ion chelating capabilities to sequester its function.^51^^,^^52^ This dual functionality raises an important question: when CueO overexpression leads to reduced copper response signals, is this effect attributable to CueO’s oxidation of Cu^+^ or to its ability to chelate and sequester copper ions? To distinguish between these two potential mechanisms, we needed to construct control strains that selectively retain or eliminate specific functions of CueO. Based on the research by Mazurenko et al.,^51^ we designed and constructed two CueO mutants: pLC25 (*BBa_J23100-*Δ*cueO*^M441Q^), a single residue mutant that retained approximately 10% of Cu^+^ oxidative activity, and pLC26 (*BBa_J23100-*Δ*cueO*^D439A, M441Q^), a double residue mutant that completely abolished Cu^+^ oxidative activity (Figure 1B). These mutations were engineered to selectively eliminate Cu^+^ oxidation capability while preserving Cu^+^ binding capacity of CueO. As shown in Figure 1C, our comparative analysis revealed that the CueO knockout strain (Cu90) and the oxidase-inactive mutant strain (Cu96) exhibited similar response curves, with Cu96 showing only a very slight reduction in response signal. Even the strain with 10% oxidative activity (Cu95) also exhibited a much higher response signal than Cu85. These results strongly indicated that while both oxidation and binding mechanisms may contribute to copper resistance, the chelation function of CueO provides minimal detoxification effect under our experimental conditions. These findings clarify CueO’s primary role in copper homeostasis.

### The reduction of Cu^2+^ to Cu^+^ occurs in the cytoplasm

**Figure 2:**
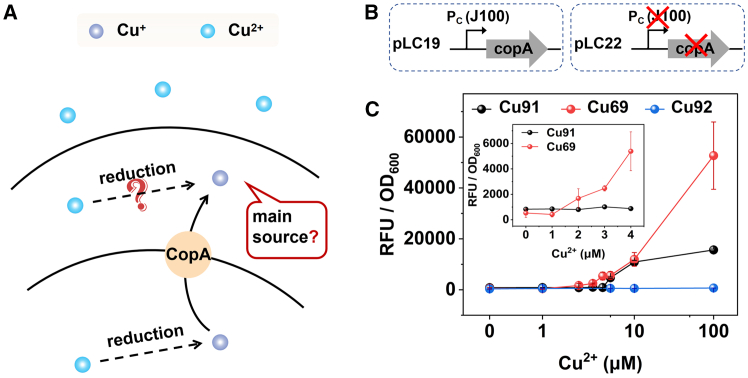
The role of CopA (A) The schematic diagram of Cu^2+^ entering the cell. (B) The schematic diagram of the genetic elements of plasmid pLC19 and pLC22. (C) The response curves of strains Cu91 (*DH5α*/pCWCu31/pLC22), Cu69 (*DH5α*/pCWCu31/pLC19) and Cu92 (*DH5α::ΔcopA/*pCWCu31/pLC22). The data of strains Cu91 and Cu69 at low concentration were taken out and displayed in the insert figure. All measurements were performed with 3 replicates; error bars denote standard deviation.

Although the results in Figure 1C indicated that Cu^2+^ was reduced to Cu^+^ upon cellular entry, the precise spatial location of this reduction within the cell remains unclear due to the presence of multiple reductases in both cytoplasm and periplasmic space of *E. coli* (Figure 2A). To clarify this question, considering the function of CopA in transporting Cu^+^ from the cytoplasm to the periplasmic space,^24^ we constructed a *copA* knockout strain (*DH5α::ΔcopA*) and an overexpression strain. The plasmid pCWCu31 and pLC22 (Figure 2B) were introduced into *DH5α::ΔcopA* to monitor Cu^+^ in the periplasmic space, resulting in strain Cu92, which was presumed to lack CopA expression. Additionally, to ensure high CopA levels in cells, we engineered a plasmid pLC19 by inserting *copA* downstream of the strong promoter *J100* (Figure 2B). Concurrently, plasmids pLC19 and pCWCu31 were co-transformed into the *DH5α*, creating strain Cu69, characterized by an exceptionally high level of CopA expression. For comparison, we designated strain Cu31(*DH5α/*pCWCu31), which exhibited a normal level of CopA, as the control. The two compatible plasmids, pCWCu31 and pLC22, were transferred into Escherichia coli *DH5α* to generate strain Cu91. The response of strain Cu91 to copper ions was also mediated by the Cus system. The response of these three strains to copper ions was depicted in Figure 2C, using identical experimental conditions as those described for testing Cu85. Notably, strain Cu92 exhibited a negligible output signal across varying concentrations of copper ions. This observation suggested that in the absence of CopA, the cell failed to respond to copper ions, implying a near absence of Cu^+^ in the periplasmic space. Consequently, it could be inferred that Cu^+^ in the periplasmic space was primarily extruded by CopA. Furthermore, it was reasonable to hypothesize that Cu^2+^ was only reduced within the cytoplasm.

Based on these findings, it is plausible to speculate that the increased expression of CopA could enhance cellular response to copper ions. We conducted experiments to compare the response of strains Cu91 and Cu69 to copper ions. As depicted in Figure 2C, the fluorescence intensity of Cu69 was significantly higher than that of Cu91, particularly at elevated copper ion concentrations. Furthermore, the subgraph of Figure 2C clearly demonstrated that the detection limit for Cu69 was lower than that for Cu91. Specifically, the detection limit was approximately ∼2 μM for Cu69 and ∼5 μM for Cu91. These findings were in complete agreement with our initial hypothesis and further indicated that Cu^+^ ions in the periplasmic space were expelled by CopA, while Cu^2+^ ions were only reduced in the cytoplasm. Therefore, CopA was essential within the CusRS two-component system, underscoring its critical role. Notably, Meydan et al.^25^ demonstrated that the copA gene utilizes programmed ribosomal frameshifting to encode the CopZ chaperone-a mechanism that potentially facilitates copper trafficking and delivery to CopA under specific physiological contexts. Future studies should focus on exploring the potential role of CopZ within the broader copper homeostasis network.

### The phosphatase function of CusS and signal crosstalk of CusR

**Figure 3:**
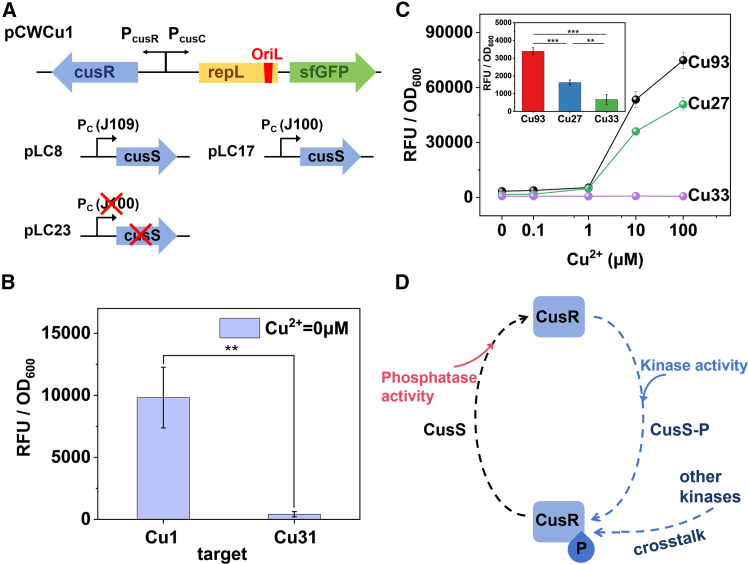
The Role of CusS on CusR (A) Schematic diagram of the genetic elements of plasmids pCWCu1, pLC8, pLC17, and pLC23. (B) The background fluorescence signal of strains Cu1 (*DH5α*/pCWCu1) and Cu31(DH5α/pCWCu31). Data are represented as mean ± SD, ∗∗: *p* < 0.01. (C) The response curves of strains Cu93(*DH5α*/pCWCu1/pLC23), Cu27 (*DH5α*/pCWCu1/pLC8) and Cu33 (*DH5α*/pCWCu1/pLC17). Inset shows the background fluorescence intensity of strains Cu93, Cu27, and Cu33. Data are represented as mean ± SD, ∗∗: *p* < 0.01, ∗∗∗: *p* < 0.001. (D) Schematic diagram of the crosstalk of CusR and the dual functionality of CusS. Measurements were performed with 3 replicates; error bars denote standard deviation.

In most two-component systems, there are two critical proteins: histidine kinase and response regulator.^29^ Typically, histidine kinase detects external signals, initiating autophosphorylation. The phosphorylated histidine kinase then interacts with the response regulator, facilitating the transfer of the phosphate group to the response regulator and activating the expression of corresponding regulatory pathways. Previous literature has noted that some histidine kinase possesses not only kinase activity but also phosphatase activity, as observed in systems such as CopRS, where CopS is believed to be able to dephosphorylate the phosphorylated CopR (CopR-P).^42^ However, there are currently no reports on whether the histidine kinase CusS possesses phosphatase activity. To substantiate the role of CusS in dephosphorylation as a phosphatase, plasmids pLC8 and pLC17 were constructed by inserting the *cusS* gene downstream of *J109* (weak promoter) and *J100* (strong promoter), respectively. Their control plasmids were the empty vector pLC23 with cusS genes removed. Meanwhile, *cusR* was constructed under the control of promoter P_*cusR*_ in pCWCu31 to create the plasmid of pCWCu1, where the expression of CusR could be activated by phosphorylated CusR(CusR-P), enabling an exogenous overexpression of CusR (Figure 3A). Subsequently, pCWCu1 was transformed into *DH5α* to create strain Cu1, pLC8, pLC17, and pLC23 were cotransformed with pCWCu1 into *DH5α*, resulting in the strains of Cu27, Cu33, and Cu93, respectively. In Cu1, the presence of copper ions can activate promoters P_*cusC*_ and P_*cusR*_, enabling the upregulation of sfGFP and CusR. Subsequently, CusR will further activate P_cusR_, ensuring a higher expression of itself. Therefore, we were able to enhance the output signal by increasing the expression of CusR in pCWCu1, and then verified whether an excess of CusS weakens the enhanced signal by detecting the fluorescence of strains with varying levels of CusS expression.

We first compared the background fluorescence signal of strains Cu1 and Cu31 in the absence of copper ions, as depicted in Figure 3B. The overnight bacterial culture was diluted with fresh LB medium supplied with ampicillin at a 1:100 ratio and incubated until the OD_600_ ≈ 0.6. The culture was then induced 5 h before analysis. The result suggested that the Cu1 strain with additional CusR expression displayed a higher background fluorescence, which was significantly different from Cu31 (*p* < 0.01), indicating that more CusR was associated with increased background signals. The observed phenomena might be due to the crosstalk phosphorylation of CusR by other kinases, such as YedV. Then the response of strains Cu93, Cu27, and Cu33 to various concentrations of copper ions was measured using the same testing procedure as those mentioned above for testing Cu85 (Figure 3C). Among these three strains, Cu93 uniquely expressed CusS on the chromosome, while Cu27 and Cu33 had additional CusS expression from a plasmid. And the promoter *J100* in Cu33 is much stronger than *J109* in Cu27. It was clear to see that Cu93 exhibited a higher fluorescence than the other two CusS overexpression-strains across all concentrations of copper ions. To emphasize baseline differences, the 0 mM Cu^2+^ data from Figure 3C was magnified and presented as an inset. The enlarged visualization revealed that the low-CusS-expressing strain Cu27 exhibited an sfGFP signal 2.4-fold higher than the high-expressing control Cu33 (*p* < 0.01). In the CusS-lacking strain Cu93, the signal intensity rose even further to 5.0-fold that of Cu33 (*p* < 0.001), with the difference between Cu93 and Cu27 remaining highly significant (*p* < 0.001). This observed gradient in response indicated that CusS abundance negatively correlated with baseline fluorescence, suggesting a potential role of CusS as a phosphatase. These findings showed that the upregulation of CusS was able to reduce the activity of P_*cusC*_ promoter, and the more CusS present, the weaker the promoter activity. The results suggest that CusS may exhibit phosphatase activity, potentially promoting the dephosphorylation of CusR-P and thus attenuating its transcriptional activation at the promoter (Figure 3D). Future studies should aim to verify the proposed phosphatase activities of CusS through direct biochemical experiments such as Phos-tag assays.

### The roles of CueO and CusCFBA on the detoxification of Cu^+^ in the periplasmic space

**Figure 4:**
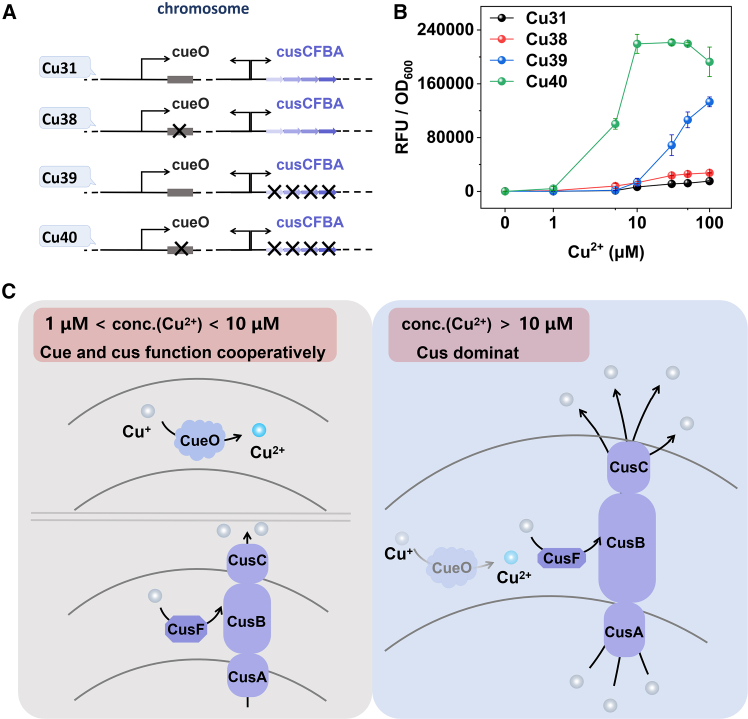
The roles of CueO and CusCFBA on copper homeostasis of E. coli (A) The schematic diagram of the genetic elements for chromosomal gene knockouts in DH5α: *DH5α::ΔcueO*, *DH5α::ΔcusCFBA,* and *DH5α::ΔcueO ΔcusCFBA*. (B) The responding curves of strains Cu31, Cu38, Cu39, and Cu40. The plasmid pCWCu31 was transformed into DH5*α*, *DH5α::ΔcueO, DH5α::ΔcusCFBA, and DH5α::ΔcueO ΔcusCFBA,* resulting in the construction of strains Cu31, Cu38, Cu39, and Cu40, respectively. All measurements were performed with 3 replicates; error bars denote standard deviation. (C) The process of Cu^+^ detoxification in the periplasmic space of E. coli under different copper ion concentrations.

In *E. coli*, in addition to CopA’s efflux of Cu^+^, there are two other pathways for copper detoxification: the oxidation of CueO and the efflux system CusCFBA. CueO oxidizes Cu^+^ to the less toxic Cu^2+^, thereby reducing the toxicity of copper ions. Conversely, CusCFBA maintains cellular homeostasis by expelling copper ions from the cytoplasm and the periplasmic space to the exterior of the cell. To elucidate the roles of CueO and CusCFBA further, we generated knockout strains of the genes *cueO, cusCFBA* and both, resulting in *DH5α::ΔcueO, DH5α::ΔcusCFBA,* and *DH5α::ΔcueO ΔcusCFBA*, respectively. The plasmid pCWCu31 was then transformed into these three host strains for the Cu^+^ response detection, yielding strains Cu38, Cu39, and Cu40, as depicted in Figure 4A.

Subsequently, we examined the fluorescence response and growth of these strains (Figures 4B and S1). The fluorescence response was measured using the same procedure as described above for testing Cu85. For measuring the growth curve, overnight bacterial cultures were diluted 1:100 with fresh LB medium supplied with ampicillin and cultured until mid-logarithmic phase (serving as the initial value of the growth curve). Different concentrations of Cu^2+^ were then added for induction, and OD_600_ was measured every 0.5 h to monitor and analyze bacterial growth. As depicted in Figure 4B, the response of the single gene knockout strains Cu38 and Cu39 to copper ions exhibited minimal variation compared to the wild-type strain Cu31 within the low concentrations between 1 μM and 10 μM. However, the double gene knockout strain Cu40 demonstrated a significant response to copper ions, with its fluorescence signal markedly higher than that of Cu38, Cu39, and Cu31. This observation suggested that at low concentrations, the Cu^+^ levels in the periplasmic space of strains with a single knockout of *cueO* or *cusCFBA* were minor, while a significant accumulation of Cu^+^ was observed in the double-knockout strain’s periplasmic. Therefore, it was plausible to infer that at low concentrations, the CueO and CusCFBA pathways could compensate for each other, with the presence of either being sufficient to detoxify Cu^+^ and maintain Cu^+^ at a low level in the periplasmic space. The growth curves depicted in Figure S1 further corroborated this conclusion. As demonstrated in Figure S1, the growth of the single knockout strain was not significantly impacted at low concentrations, which suggests that the presence of either the CueO or CusCFBA pathways is adequate to mitigate the cellular toxicity caused by copper ions. Consequently, it can be inferred that CueO and CusCFBA pathways can be substituted for each other at low concentrations. However, at elevated concentrations, the growth of all knockout strains exhibited various degrees of inhibition, whereas the growth of wild type strains remained unaffected. This observation indicates that, at high concentrations, CueO or CusCFBA alone are insufficient to counteract the cytotoxic effects of copper ions. Thus, at high concentrations, the CueO and CusCFBA pathways operate synergistically to detoxify copper ions.

At elevated concentrations, the response curves of Cu31 and Cu38 were observed to be similar, while the fluorescence signal of Cu38 being slightly higher than that of Cu31 (Figures 4B and S2). This indicated that, at high copper concentrations, the absence of CueO did not lead to a significant change in the Cu^+^ level in the periplasmic space when CusCFBA was present, thus indicating that CusCFBA might play a dominant role in the detoxification of Cu^+^ in the periplasmic space. Conversely, the response curves of Cu31 and Cu39 exhibited substantial differences at high concentrations, with the fluorescence signal of Cu39 being significantly higher than that of Cu31 (Figure 4B). This observation suggested that the knockout of the *cusCFBA* genes led to a considerable accumulation of Cu^+^ in the periplasmic space, further demonstrating the importance of *cusCFBA*. Consequently, we speculated that the CusCFBA copper efflux pathway played a central role in the detoxification of copper ions at elevated concentrations.

In conclusion, concerning the detoxification process of Cu^+^ within the periplasmic space, at very low extracellular Cu^2+^ concentrations (<1 μM), the two-component system remained inactive. As the concentration increased (1 μM < extracellular Cu^2+^ concentration <10 μM), the CueO oxidation and CusCFBA expulsion mechanisms acted synergistically and were interchangeable in maintaining periplasmic Cu^+^ equilibrium. In other words, the presence of one system was sufficient to sustain low Cu^+^ levels in the periplasm when the other was absent (Figure 4C left). At even higher extracellular Cu^2+^ concentrations (>10 μM), the CusCFBA expulsion mechanism predominated. This observation suggested that CueO had a limited capacity for Cu^+^ detoxification in the periplasm, whereas CusCFBA assumed a more significant role in this detoxification process (Figure 4C right).

### The interrelationship and synergy between the cue system and the cus system

**Figure 5:**
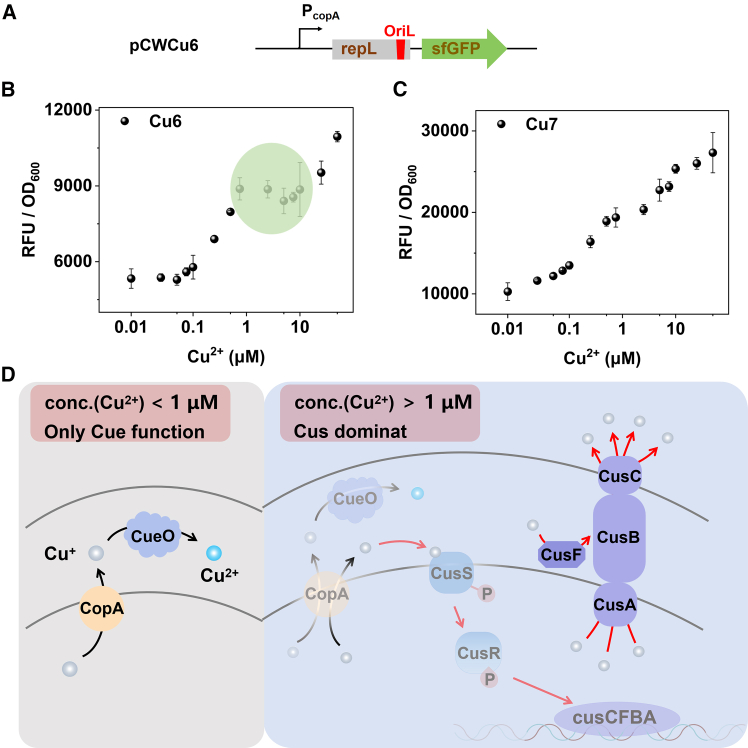
The response of the P_copA_ promoter in the cytoplasmic (A) The schematic diagram of the genetic elements of plasmid pCWCu6. (B and C) The response curves of strains Cu6 (*DH5α/*pCWCu6) and Cu7 (*DH5α::ΔcopA*/pCWCu6). All measurements were performed with 3 replicates; error bars denote standard deviation. (D) The diagram illustrating the dynamic detoxification of Cu^+^ within the cytoplasm and periplasmic space of E. coli, highlighting the interplay between the *cue* and *cus* regulatory systems.

To systematically assess the C*ue* and C*us* systems, we inserted the fluorescent protein gene *sfGFP* and the signal amplification module *repL* downstream of the promoter P_*copA*_ to generate the plasmid pCWCu6 for the detection of cytoplasmic Cu^+^ levels (Figure 5A). pCWCu6 was transformed into the host strains of *DH5α* and *DH5α::ΔcopA*, resulting in the creation of strains Cu6 and Cu7. Subsequently, the responses of strains Cu6 and Cu7 were evaluated using the same testing procedure as described above for testing Cu85. As depicted in Figure 5B, it was observed that the fluorescence intensity of strain Cu6 slightly decreased within the copper ion concentration range of approximately 1 μM–10 μM, indicating a reduction of Cu^+^ in the cytoplasmic. Whereas, the decrease in fluorescence almost vanished in the strain of Cu7 when the *copA* gene was knockout (Figure 5C). Concurrently, we noted that the Cus system initiated its response at a Cu^2+^ concentration of 1 μM (Figures 4B and S2). Based on these observations, the Cus system was likely activated at ∼1 μM, leading to the efflux of Cu^+^ ions, which resulted in a reduction of Cu^+^ in the cytoplasm and a subsequent decrease in the fluorescence signal. As a result, when the extracellular Cu^2+^ concentration ranged between 1 μM and 10 μM, the cytoplasmic Cu^+^ concentration remained relatively stable despite the increase in extracellular Cu^2+^. However, in the absence of CopA, Cu^+^ could not be expelled into the periplasmic space, the Cus system failed to activate, eliminating this behavior and exhibiting a more consistent linearity.

The detection limit for Cue-based copper ion biosensors, as reported in prior literature,^16^ has consistently been relatively low. Our experimental results were consistent with these findings. As illustrated in Figures 5B and 5C, the promoter P_*copA*_ was activated at a Cu^2+^ concentration of 0.1 μM in strain Cu6 and 0.01 μM in the *copA*-deficient strain Cu7. Both values were lower than the concentration at which the promoter P_*cusC*_ began to activate in Cu31 and Cu40. Consequently, we proposed that the Cue system was the initial responder to low concentrations of Cu^2+^. When the concentration of extracellular Cu^2+^ was lower than 1 μM, there was a minimal presence of Cu^+^ in the periplasm, insufficient to activate the Cus system’s response (Figure 5D). As the Cu^2+^ concentration increased, CopA progressively elevated, resulting in an increased transfer of Cu^+^ into the periplasmic space. Upon exceeding an extracellular Cu^2+^ concentration of 1 μM, the Cus system was activated, significantly enhancing the expression of CusCFBA, which actively expelled Cu^+^ from the cytoplasm and periplasmic space (Figure 5D). Therefore, the response of *E. coli* cells to Cu^2+^ could be sequenced as follows: first, Cu^2+^ was reduced to Cu^+^ in the cytoplasm, then the Cue system was activated, inducing the upregulation of CopA, which led to the expulsion of Cu^+^ into the periplasmic space. Subsequently, the Cus system was activated to export Cu^+^ out of the cell to maintain homeostasis.

### Concluding remarks

**Figure 6:**
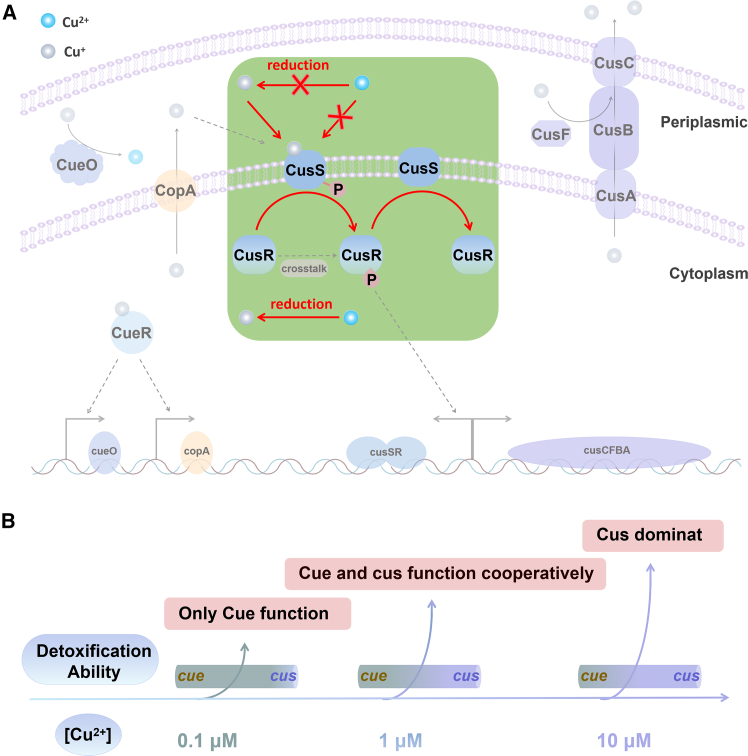
Copper ion tolerance system in E. coli: cue system and cus system (A) Schematic figure of the copper efflux system, cue and cus systems in E. coli. (B) Response of the Escherichia coli cue and cus system to different copper ion concentrations.

In conclusion, our study in this work has unveiled several crucial aspects of copper homeostasis, providing both fundamental insights and practical applications. We comprehensively explored the response of copper homeostasis in *E. coli* through systematic and synthetic biology approaches, investigating in detail the entire process of *E. coli*’s response to and detoxification of copper ions (Figure 6A). Initially, through the overexpression of CueO, we confirmed that the Cus system exhibits a highly sensitive response to Cu^+^ with negligible response to Cu^2+^. Subsequently, by modulating the expression level of the *copA* gene, we discovered that the reduction of Cu^2+^ to Cu^+^ predominantly occurs in the cytoplasm, while Cu^+^ in the periplasmic space was principally derived from CopA exporting it out of the cytoplasm. Further investigations revealed that CusR exhibited signal crosstalk, leading to a baseline expression, which could be mitigated by an optimal concentration of CusS. Furthermore, our results also demonstrated that CusS exhibited both kinase and phosphatase activities. In the absence of copper ions, CusS primarily functions as a phosphatase, facilitating the dephosphorylation of CusR-P. Conversely, in the presence of Cu^+^, CusS predominantly acted as a kinase, phosphorylating CusR to CusR-P.

Comparing the single and double knockout strains of the *cueO* or *cusCFBA* genes with the wild-type strain revealed that, at low copper ion concentrations, CusCFBA and CueO cooperated in Cu^+^ detoxification within the periplasmic space and were functionally interchangeable. In contrast, CusCFBA assumed a dominant role at high concentrations due to the limited detoxification capacity of CueO. Finally, upon comparing the Cue and Cus systems, we inferred that the Cus system was activated only when the Cue system responded to low concentrations of copper ions. According to the results above, we can systematically describe the response and metabolism of Cu^2+^ within *E. coli* cells as follows (Figure 6B): Upon entering into cytoplasm, Cu^2+^ is reduced to Cu^+^ by intracellular reductases. The cue system is then activated, leading to the upregulation of CueO and CopA. Subsequently, with the increase of extracellular Cu^2+^ (∼0.1 μM), CopA starts to efflux Cu^+^ from the cytoplasm into the periplasmic space. When the extracellular Cu^2+^ concentration is relatively high (1–10 μM), the upregulation of CopA leads to an accumulation of Cu^+^ in the periplasm, which activates the Cus system. At this stage, the Cu^+^ in the cytoplasm is primarily eliminated through the combined efflux actions of CopA and CusCFBA, while the detoxification of Cu^+^ in the periplasmic space is achieved through both oxidation by CueO and efflux by CusCFBA. Whereas, with the extracellular Cu^2+^ concentration increases further (>10 μM), CusCFBA becomes the primary contributor to periplasmic space Cu^+^ detoxification due to the limited detoxification capacity of CueO. This mechanism demonstrates that the cell employs a dual strategy to combat copper ion toxicity: the Cue system dominates at low copper ion concentrations, whereas the Cus system becomes more important at medium to high concentrations. At very high concentrations, both systems may operate in tandem. This investigation elucidated the relationship between the two copper ion response systems in *E. coli*. The findings in this study not only enrich our understanding of bacterial two-component systems but are also valuable for researchers working on microbiology, biochemistry, and synthetic biology, particularly those focused on metal homeostasis, signal transduction, and bacterial adaptation mechanisms. Future work based on these findings could lead to innovative solutions in environmental protection, medical treatments, and biotechnology applications.

### Limitations of the study

Although this study offers new insights into the copper homeostasis regulatory network in *E. coli*, several limitations remain to be addressed in future work. First, *in vivo* evidence remains limited. Our conclusions rely largely on phenotypic analyses using reporter systems and gene-knockout strains. The direct, real-time monitoring of key processes-such as the phosphorylation states of CusS/CusR and the dynamic distribution of intracellular Cu^+^-has not been achieved. Second, the physiological relevance of the experimental conditions requires further validation. Although a broad range of copper concentrations was tested, these may not fully mimic the copper stress conditions encountered in natural environments or host systems. Thus, the biological significance of these findings warrants additional investigation. Third, certain molecular mechanisms demand deeper characterization. For instance, the structural basis of how CusS responds to Cu^+^ and switches between kinase and phosphatase activities remains unclear. Furthermore, potential cross-regulation between the Cue and Cus systems at the transcriptional level has yet to be explored. Finally, the practical applications of this research need experimental support. Although potential uses in environmental bioremediation and biotechnology are proposed, functional validation, such as assessing the performance and detoxification efficiency of engineered strains in real copper-contaminated settings still lacking. Future studies focusing on these aspects will help to refine our understanding of bacterial copper homeostasis and facilitate its translation into practical applications.

## Resource availability

### Lead contact

Further information and requests should be directed to and will be fulfilled by the lead contact, Xiaona Fang (fangxn482@nenu.edu.cn).

### Materials availability

This study did not generate new materials.

### Data and code availability

• All data reported in this article will be shared by the lead contact upon reasonable request.
• This study did not generate new code. Any additional information required to reanalyze the data reported in this article is available from the lead contact upon request.
• Any additional information required to reanalyze the data reported in this article is available from the lead contact upon request.

## Acknowledgments

X.F., Z.C., Y.F. and J.L. were supported by the 10.13039/501100001809National Natural Science Foundation of China (no. 32171245), the 10.13039/501100012166National Key Research and Development Program of China (2024YFA 0919600) and the 10.13039/501100012226Fundamental Research Funds for the Central Universities (no. 2412022QD011). Z.C., X.F., and J.L. acknowledge the support from the 10.13039/501100010211Jilin Provincial Department Education, the Key Laboratory of Nanobiosensing and Nanobioanalysis at Universities of Jilin Province (China), and the Analysis and Testing Center of Northeast Normal University (China). We thank Dr. Neal K. Devaraj for providing the pSB1C3-pT3-tetR-sfGFP plasmid.

## Author contributions

X.F. designed and supervised the projects. Z.C. and Y.F. engineered the strains, performed all the experiments and data analysis. J.L. constructed some of the original plasmids. Z.C., X.F., and Y.F. wrote the article with comments from all authors. X.F., E.W., and J.W. discussed and interpreted the results, reviewed and edited the article.

## Declaration of interests

The authors declare no conflicts of interest.

## STAR★Methods

### Key resources table

**Table:** 

REAGENT or RESOURCE	SOURCE	IDENTIFIER
**Bacterial and virus strains**		

*Escherichia coli DH5a*	Laboratory	N/A
*Escherichia coli DH5a*_*pCwCu1*	Laboratory	N/A
*Escherichia coli* DH5a_pCwCu6	This paper	N/A
*Escherichia coli* DH5a_Δ*copA*_pCwCu6	This paper	N/A
*Escherichia coli* DH5a_pCwCu1/pLC8	Laboratory	N/A
*Escherichia coli* DH5a_pCwCu31	Laboratory	N/A
*Escherichia coli* DH5a_pCwCu1/pLC17	This paper	N/A
*Escherichia coli* DH5a_Δ*cueO*_pCwCu31	This paper	N/A
*Escherichia coli* DH5a_Δ*cusCFBA*_pCwCu31	This paper	N/A
*Escherichia coli* DH5a_Δ*cueO* Δ*cusCFBA*_pCwCu31	This paper	N/A
*Escherichia coli* DH5a_Δ*copA*_pCwCu31	This paper	N/A
*Escherichia coli* DH5a_pCwCu31/pLC19	This paper	N/A
*Escherichia coli* DH5a_Δ*cusCFBA*_pCwCu31/pLC20	This paper	N/A
*Escherichia coli* DH5a_Δ*cusCFBA*_pCwCu31/pLC21	This paper	N/A
*Escherichia coli* DH5a_Δ*cusCFBA*_pCwCu31/pLC22	This paper	N/A
*Escherichia coli* DH5a_Δ*copA*_pCwCu31/pLC22	This paper	N/A
*Escherichia coli* DH5a_pCwCu1/pLC23	This paper	N/A
*Escherichia coli* DH5a_Δ*cusCFBA*_pCwCu31/pLC25	This paper	N/A
*Escherichia coli* DH5a_Δ*cusCFBA*_pCwCu31/pLC26	This paper	N/A

**Chemicals, peptides, and recombinant proteins**		

Tryptone	Solarbio	Cat#T8940
yeast extract	Solarbio	Cat#Y8020
agar	Solarbio	Cat#A8190
NaCl	Solarbio	Cat#S8210
ampicillin	Solarbio	Cat# A1170
kanamycin	Solarbio	Cat# K1030
spectinomycin	Solarbio	Cat#S8040
CuCl_2_.2H_2_O	Sigma Aldrich	Cat#C3279
PBS,1X	Servicebio	Cat#G4202

**Oligonucleotides**		

Primers for strain construction and test, see Table S3	This paper	N/A

**Software and algorithms**		

Prism 8	GraphPad Software	https://www.graphpad.com/

### Experimental model and study participant details

#### Bacterial strains, reagents and growth medium

Plasmid construction, biosensor detection and gene knockout were all performed in host strain of *E. coli DH5α*. Cells were cultured in fresh Lysogeny Broth (LB) medium supplemented with the appropriate antibiotics. The LB medium was prepared by dissolving 1 g of yeast extract, 2 g of tryptone, and 2 g of sodium chloride in 200 ml of deionized water, followed by autoclaving at 121 °C for 15 minutes. The antibiotic concentrations used for strain selection were as follows: 50 μg/ml for ampicillin (Cat: A1170), 50 μg/ml for kanamycin (Cat: K1030), 50 μg/ml for spectinomycin (Cat: S8040), and 10 μg/ml for chloramphenicol (Cat: C8050), as applicable. Tryptone, yeast extract, sodium chloride, agar, kanamycin, ampicillin, spectinomycin and chloramphenicol were purchased from Solarbio. CuCl_2_⋅2H_2_O were purchased from Sigma Aldrich. The phosphate buffered saline (PBS, 1X) were purchased from Servicebio. pSB1C3-pT3-tetR-sfGFP was a gift from Neal Devaraj (Addgene plasmid #140871; http://n2t.net/addgene:140871; RRID: Addgene_140871).^53^

### Method details

#### Testing conditions

For the characterization of biosensors, all measurements were performed with 3 replicates (the practice of randomly selecting three independent parallel bacterial plaques for each experiment and performing identical experimental operations for each plaque), and single colonies were selected and inoculated into 5 ml of LB medium supplemented with the appropriate antibiotic overnight (about 14∼16 hours) at 37 °C and 250 r.p.m. in a shaker (IS-RSD3). Then the next day, the cells were diluted (1:100) in fresh LB medium and continuous shaken (250 r.p.m.) at 37°C until the optical density at 600 nm (OD_600_) reached 0.5∼0.7 (about 2.5∼3 h). Subsequently, a series of CuCl_2_ solution with different concentration gradient were added to the bacteria cultures and incubated for approximately 5 h for detection. The appropriate volume of CuCl_2_ dilutions were added to achieve final concentration of 0.1, 1, 2, 3, 4, 5, 10, 100 μM. Afterward, 500 μl bacteria cultures were collected and centrifuged for 1 min at 12000 g, then washed and resuspended in 1.5 ml of phosphate-buffered saline (PBS). Finally, 200 μl of diluted samples were added to a 96 well plate for measurement using a microplate reader (TECAN, Spark). The absorbance at 600 nm (OD_600_) was measured to obtain the cell density. And the excitation wavelength was set to 475 nm, meanwhile the fluorescence signal of superfold green fluorescent protein (sfGFP) was recorded between 505 nm and 560 nm. For the medium background, PBS was measured in every experiment, and the wild-type *DH5α* bacteria was measured as a negative control.

#### Construction of plasmid and bacterial strains

We employed standard molecular biology methods to construct all plasmids utilized in this study. Details regarding the plasmids, strains, and primers used were summarized in Tables S1–S3, with confirmation obtained through sequencing (Sangon, shanghai).

For observing the response of Cus system to copper ion, the promoter with *cusR* gene (*cusR*-P_*cusR*_-P_*cusC*_) and *repL-sfGFP* genes fragment were amplified from the *E. coli* K12 MG1655 chromosome and pMT012 (our lab) by polymerase chain reaction (PCR) separately,^49^ and then they were put together by T4 ligase to construct plasmid pCWCu1 (*cusR*-P_*cusR*_-P_*cusC*_-*repL-sfGFP*). And the *cusR* gene was knockout by inverse PCR to construct plasmid pCWCu31 (P_cusR_-P_*cusC*_*-repL-sfGFP*). For tuning CusS’s expression, the promoter *BBa_J23109* and *cusS* gene were amplified from pLC4 (our lab) and the *E. coli* K12 MG1655 chromosome separately via PCR, and they were linked to obtain plasmid pLC8 (*BBa_J23109-cusS*). Next, *copA* gene in *E. coli* K12 MG1655 genome was introduced to pLC8, and we replaced its promoter with *BBa_J23100* by PCR to construct plasmid pLC17 (*BBa_J23100-cusS-copA*). To establish a control for the system, we generated plasmid pLC23 by precisely deleting the *BBa_J23100* promoter and cusS gene from pLC17.

To tune the expression level of CopA, the *cusS* gene was knockout between the promoter *BBa_J23100* and *copA* gene at pLC17 by inverse PCR to obtain plasmid pLC19 (*BBa_J23100-copA*).

Plasmid pLC22 was created by complete deletion of both the *BBa_J23100* promoter and *copA* coding sequence from pLC19 to serve as a null control. For overexpressing CueO, the *copA* gene in pLC19 was replaced with the *cueO* gene sequence, which was from the *E. coli* K12 MG1655 genome, via PCR, thus plasmid pLC20 (*BBa_J23100-cueO*) was constructed. Then, the corresponding control plasmid pLC21 was generated by precisely deleting the *BBa_J23100* promoter and the entire *CueO* coding sequence.

Finally, regarding the response of Cue system, the promoter P_*copA*_, was amplified from pMT010 (our lab), replaced promoter P_*ars-*84_ in pMT012 by PCR to construct plasmid pCWCu6 (P_*copA*_*-repL-sfGFP*).

To explore the role of the genes involved in the CusRS system, we modified the *E. coli DH5α* strain using λ red homologous recombination method. We replaced *cueO* and *cusCFBA* genes on the chromosome with *kanamycin* and *spectinomycin* resistance gene, respectively. Thus we obtained knockout strain *E. coli DH5α::ΔcueO*, *E. coli DH5α::ΔcusCFBA* and double knockout strain *E. coli DH5α::ΔcueO ΔcusCFBA*. Next we replaced *copA* gene on the chromosome with a chloramphenicol resistance gene to generate the knockout strain *E. coli DH5α::ΔcopA*.

### Quantification and statistical analysis

Data were analyzed using GraphPad Prism 8.0. Between-group differences were evaluated by unpaired two tailed Student’s t-tests. One-way analysis of variance (ANOVA) followed by Tukey's multiple comparisons was used to analyze statistically significant differences between more than two groups. All experiments were performed with three independent replicates, and data are presented as mean ± standard deviation (SD). Statistical significance was defined as follows: ∗∗P < 0.01 and ∗∗∗P < 0.001.

## References

[1] Andrei A., Öztürk Y., Khalfaoui-Hassani B., Rauch J., Marckmann D., Trasnea P.-I., Daldal F., Koch H.-G. (2020). Cu Homeostasis in Bacteria: The Ins and Outs. Membranes.

[2] Chen J., Jiang Y., Shi H., Peng Y., Fan X., Li C. (2020). The molecular mechanisms of copper metabolism and its roles in human diseases. Pflugers Arch..

[3] Ladomersky E., Petris M.J. (2015). Copper tolerance and virulence in bacteria. Metallomics.

[4] Chen L., Min J., Wang F. (2022). Copper homeostasis and cuproptosis in health and disease. Signal Transduct. Target. Ther..

[5] Festa R.A., Thiele D.J. (2011). Copper: An essential metal in biology. Curr. Biol..

[6] Yang S., Li Y., Zhou L., Wang X., Liu L., Wu M. (2024). Copper homeostasis and cuproptosis in atherosclerosis: metabolism, mechanisms and potential therapeutic strategies. Cell Death Discov..

[7] Xue Q., Kang R., Klionsky D.J., Tang D., Liu J., Chen X. (2023). Copper metabolism in cell death and autophagy. Autophagy.

[8] Li C., Li Y., Ding C. (2019). The Role of Copper Homeostasis at the Host-Pathogen Axis: From Bacteria to Fungi. Int. J. Mol. Sci..

[9] Hofmann L., Hirsch M., Ruthstein S. (2021). Advances in Understanding of the Copper Homeostasis in Pseudomonas aeruginosa. Int. J. Mol. Sci..

[10] Giachino A., Waldron K.J. (2020). Copper tolerance in bacteria requires the activation of multiple accessory pathways. Mol. Microbiol..

[11] Das S., Dash H.R., Chakraborty J. (2016). Genetic basis and importance of metal resistant genes in bacteria for bioremediation of contaminated environments with toxic metal pollutants. Appl. Microbiol. Biotechnol..

[12] Rensing C., Grass G. (2003). Escherichia colimechanisms of copper homeostasis in a changing environment. FEMS Microbiol. Rev..

[13] Staehlin B.M., Gibbons J.G., Rokas A., O’Halloran T.V., Slot J.C. (2016). Evolution of a heavy metal homeostasis/resistance island reflects increasing copper stress in Enterobacteria. Genome Biol. Evol..

[14] Wen Q., Liu X., Wang H., Lin J. (2014). A versatile and efficient markerless gene disruption system for Acidithiobacillus thiooxidans: application for characterizing a copper tolerance related multicopper oxidase gene. Environ. Microbiol..

[15] Bittner L.-M., Kraus A., Schäkermann S., Narberhaus F. (2017). The Copper Efflux Regulator CueR Is Subject to ATP-Dependent Proteolysis in Escherichia coli. Front. Mol. Biosci..

[16] Wang W., Jiang F., Wu F., Li J., Ge R., Li J., Tan G., Pang Y., Zhou X., Ren X. (2019). Biodetection and bioremediation of copper ions in environmental water samples using a temperature-controlled, dual-functional Escherichia coli cell. Appl. Microbiol. Biotechnol..

[17] Mazurenko I., Adachi T., Ezraty B., Ilbert M., Sowa K., Lojou E. (2022). Electrochemistry of copper efflux oxidase-like multicopper oxidases involved in copper homeostasis. Curr. Opin. Electrochem..

[18] Tree J.J., Ulett G.C., Hobman J.L., Constantinidou C., Brown N.L., Kershaw C., Schembri M.A., Jennings M.P., McEwan A.G. (2007). The multicopper oxidase (CueO) and cell aggregation in Escherichia coli. Environ. Microbiol..

[19] Djoko K.Y., Chong L.X., Wedd A.G., Xiao Z. (2010). Reaction mechanisms of the multicopper oxidase CueO from Escherichia coli support its functional role as a cuprous oxidase. J. Am. Chem. Soc..

[20] Grass G., Rensing C. (2001). CueO Is a Multi-copper Oxidase That Confers Copper Tolerance in Escherichia coli. Biochem. Biophys. Res. Commun..

[21] Roberts S.A., Weichsel A., Grass G., Thakali K., Hazzard J.T., Tollin G., Rensing C., Montfort W.R. (2002). Crystal structure and electron transfer kinetics of CueO, a multicopper oxidase required for copper homeostasis in Escherichia coli. Proc. Natl. Acad. Sci. USA.

[22] Kataoka K., Komori H., Ueki Y., Konno Y., Kamitaka Y., Kurose S., Tsujimura S., Higuchi Y., Kano K., Seo D., Sakurai T. (2007). Structure and Function of the Engineered Multicopper Oxidase CueO from Escherichia coli—Deletion of the Methionine-Rich Helical Region Covering the Substrate-Binding Site. J. Mol. Biol..

[23] Singh S.K., Roberts S.A., McDevitt S.F., Weichsel A., Wildner G.F., Grass G.B., Rensing C., Montfort W.R. (2011). Crystal Structures of Multicopper Oxidase CueO Bound to Copper(I) and Silver(I). J. Biol. Chem..

[24] González-Guerrero M., Argüello J.M. (2008). Mechanism of Cu+-transporting ATPases: soluble Cu+ chaperones directly transfer Cu+ to transmembrane transport sites. Proc. Natl. Acad. Sci. USA.

[25] Meydan S., Klepacki D., Karthikeyan S., Margus T., Thomas P., Jones J.E., Khan Y., Briggs J., Dinman J.D., Vázquez-Laslop N., Mankin A.S. (2017). Programmed Ribosomal Frameshifting Generates a Copper Transporter and a Copper Chaperone from the Same Gene. Mol. Cell.

[26] Rensing C., Fan B., Sharma R., Mitra B., Rosen B.P. (2000). CopA: An Escherichia coli Cu(I)-translocating P-type ATPase. Proc. Natl. Acad. Sci. USA.

[27] Besaury L., Pawlak B., Quillet L. (2016). Expression of copper-resistance genes in microbial communities under copper stress and oxic/anoxic conditions. Environ. Sci. Pollut. Res. Int..

[28] Padilla-Benavides T., George Thompson A.M., McEvoy M.M., Argüello J.M. (2014). Mechanism of ATPase-mediated Cu+ Export and Delivery to Periplasmic Chaperones. J. Biol. Chem..

[29] Fu B., Sengupta K., Genova L.A., Santiago A.G., Jung W., Krzemiński Ł., Chakraborty U.K., Zhang W., Chen P. (2020). Metal-induced sensor mobilization turns on affinity to activate regulator for metal detoxification in live bacteria. Proc. Natl. Acad. Sci. USA.

[30] Ravikumar S., Pham V.D., Lee S.H., Yoo I.-K., Hong S.H. (2012). Modification of CusSR bacterial two-component systems by the introduction of an inducible positive feedback loop. J. Ind. Microbiol. Biotechnol..

[31] Affandi T., McEvoy M.M. (2019). Mechanism of metal ion-induced activation of a two-component sensor kinase. Biochem. J..

[32] Chen D., Zhao Y., Qiu Y., Xiao L., He H., Zheng D., Li X., Yu X., Xu N., Hu X. (2019). CusS-CusR Two-Component System Mediates Tigecycline Resistance in Carbapenem-Resistant Klebsiella pneumoniae. Front. Microbiol..

[33] Zahid N., Zulfiqar S., Shakoori A.R. (2012). Functional analysis of cus operon promoter of Klebsiella pneumoniae using E. coli lacZ assay. Gene.

[34] Shafer C.M., Tseng A., Allard P., McEvoy M.M. (2021). Strength of Cu-efflux response in Escherichia coli coordinates metal resistance in Caenorhabditis elegans and contributes to the severity of environmental toxicity. J. Biol. Chem..

[35] Rismondo J., Große C., Nies D.H. (2023). The Sensory Histidine Kinase CusS of Escherichia coli Senses Periplasmic Copper Ions. Microbiol. Spectr..

[36] Delmar J.A., Su C.-C., Yu E.W. (2014). Bacterial Multidrug Efflux Transporters. Annu. Rev. Biophys..

[37] Kim E.-H., Rensing C., McEvoy M.M. (2010). Chaperone-mediated copper handling in the periplasm. Nat. Prod. Rep..

[38] Delmar J.A., Su C.C., Yu E.W. (2015). Heavy metal transport by the CusCFBA efflux system. Protein Sci..

[39] Vergnes A., Henry C., Grassini G., Loiseau L., El Hajj S., Denis Y., Galinier A., Vertommen D., Aussel L., Ezraty B. (2022). Periplasmic oxidized-protein repair during copper stress in E. coli: A focus on the metallochaperone CusF. PLoS Genet..

[40] Affandi T., Issaian A.V., McEvoy M.M. (2016). The Structure of the Periplasmic Sensor Domain of the Histidine Kinase CusS Shows Unusual Metal Ion Coordination at the Dimeric Interface. Biochemistry.

[41] Casino P., Miguel-Romero L., Marina A. (2014). Visualizing autophosphorylation in histidine kinases. Nat. Commun..

[42] Novoa-Aponte L., Xu C., Soncini F.C., Argüello J.M., Ellermeier C.D. (2020). The Two-Component System CopRS Maintains Subfemtomolar Levels of Free Copper in the Periplasm of Pseudomonas aeruginosa Using a Phosphatase-Based Mechanism. mSphere.

[43] Kim E.-H., Nies D.H., McEvoy M.M., Rensing C. (2011). Switch or Funnel: How RND-Type Transport Systems Control Periplasmic Metal Homeostasis. J. Bacteriol..

[44] Outten F.W., Outten C.E., Hale J., O'Halloran T.V. (2000). Transcriptional Activation of an Escherichia coliCopper Efflux Regulon by the Chromosomal MerR Homologue, CueR. J. Biol. Chem..

[45] Gudipaty S.A., Larsen A.S., Rensing C., McEvoy M.M. (2012). Regulation of Cu(I)/Ag(I) efflux genes in Escherichia coli by the sensor kinase CusS. FEMS Microbiol. Lett..

[46] Urano H., Umezawa Y., Yamamoto K., Ishihama A., Ogasawara H. (2015). Cooperative regulation of the common target genes between H_2_O_2_-sensing YedVW and Cu^2+^-sensing CusSR in Escherichia coli. Microbiology.

[47] Grass G., Rensing C. (2001). Genes involved in copper homeostasis in Escherichia coli. J. Bacteriol..

[48] Ishihara J.I., Mekubo T., Kusaka C., Kondo S., Oiko R., Igarashi K., Aiba H., Ishikawa S., Ogasawara N., Oshima T., Takahashi H. (2023). A critical role of the periplasm in copper homeostasis in Gram-negative bacteria. Biosystems.

[49] Li J., Cui M., Zhao J., Wang J., Fang X. (2023). A self-amplifying plasmid based ultrasensitive biosensor for the detection of As(Ⅲ) in water. Biosens. Bioelectron..

[50] Bazzi W., Abou Fayad A.G., Nasser A., Haraoui L.-P., Dewachi O., Abou-Sitta G., Nguyen V.-K., Abara A., Karah N., Landecker H. (2020). Heavy Metal Toxicity in Armed Conflicts Potentiates AMR in A. baumannii by Selecting for Antibiotic and Heavy Metal Co-resistance Mechanisms. Front. Microbiol..

[51] Contaldo U., Savant-Aira D., Vergnes A., Becam J., Biaso F., Ilbert M., Aussel L., Ezraty B., Lojou E., Mazurenko I. (2024). Methionine-rich domains emerge as facilitators of copper recruitment in detoxification systems. Proc. Natl. Acad. Sci. USA.

[52] Contaldo U., Santucci P., Vergnes A., Leone P., Becam J., Biaso F., Ilbert M., Ezraty B., Lojou E., Mazurenko I. (2025). How the Larger Methionine-Rich Domain of CueO from Hafnia alvei Enhances Cuprous Oxidation. JACS Au.

[53] Niederholtmeyer H., Chaggan C., Devaraj N.K. (2018). Communication and quorum sensing in non-living mimics of eukaryotic cells. Nat. Commun..

