# Supplementary material for: Systematic and synthetic biology insights into copper homeostasis in Escherichia coli
Source: iScience. 2025 Oct 6;28(11):113715. doi: 10.1016/j.isci.2025.113715 (PMC12589874; doi:10.1016/j.isci.2025.113715)
Supplement: Document S1. Figures S1 and S2 and Tables S1–S3 [file mmc1.pdf]

iScience, Volume *28*

Supplemental information

Systematic and synthetic biology insights into copper homeostasis in Escherichia coli

Zhiqiang Chen, Yu Fu, Jiajia Li, Jin Wang, and Xiaona Fang

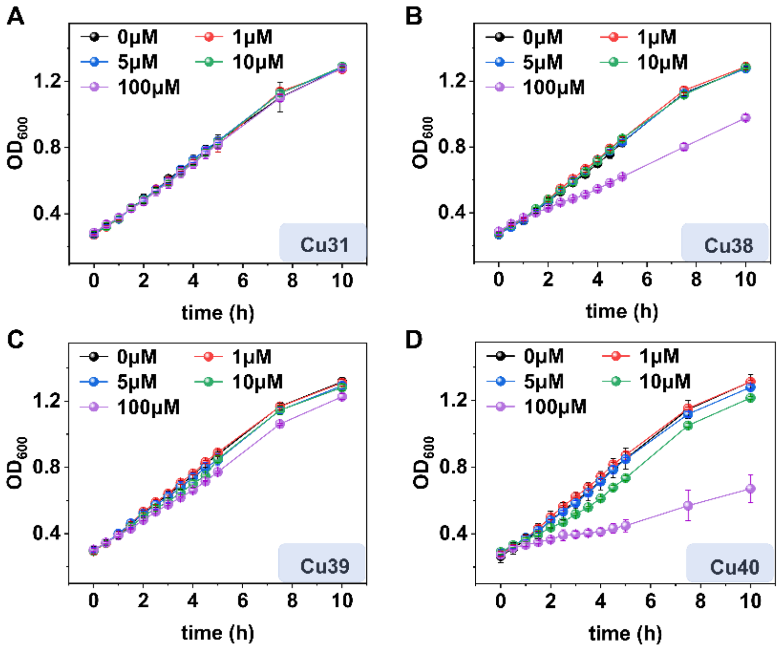

Figure S1. Growth Kinetics of Different Bacteria Strains Under Copper Stress. (A-D) The growth curves of strains Cu31, Cu38, Cu39 and Cu40 under different concentrations of Cu^{2+}. All measured with 3 replicates; error bars denoted standard deviation.

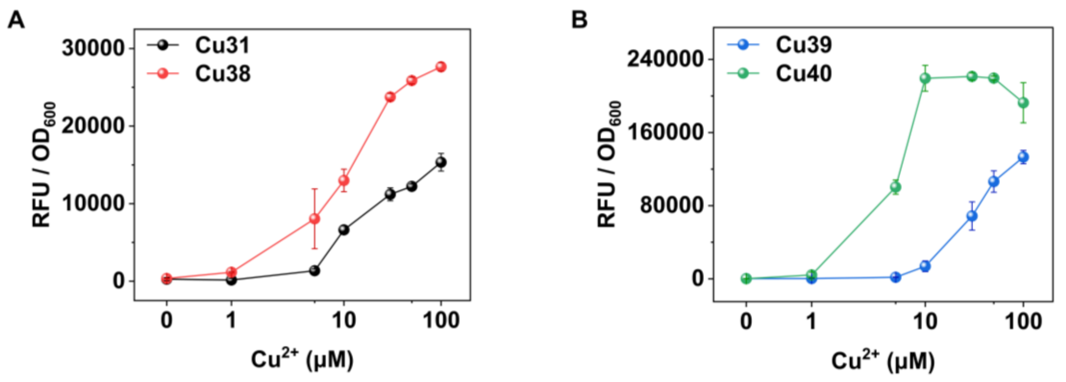

Figure S2. Comparative Analysis of Fluorescence Response in Bacterial Strains under Varying Copper Ion Stress. (A) The responding curves of strains Cu31 and Cu38. (B) The responding curves of strains Cu39 and Cu40. All measured with 3 replicates; error bars denoted standard deviation.

Table S1: Plasmid information.

Name	Plasmid information	Ori	Resistance gene	Source
pSB1C3-pT3-tetR-sfGFP	tetR-sfGFP-fusion protein	pBR322	cm^r (10μg/ml)	Addgene (140871)
pXW109hg	pSB4A3 carrying J109-32merR-t-PmerT-Amp30E-Amp31E11A-30rinA-ASV-t	pSC101	amp^r (50μg/ml)	1
pXW109ABS84	pXWAmp30E-gfp encoded by J109-30arsR-t-ParsR-ABS84	p15A	kan^r (50μg/ml)	1
pLC8	BBaJ23109-cusS	pBR322	cm^r (10μg/ml)	2
pLC17	BBaJ23100-cusS-copA	pBR322	cm^r (10μg/ml)	2
pLC19	BBaJ23100-copA	pBR322	cm^r (10μg/ml)	This study
pLC20	BBaJ23100-cueO	pBR322	cm^r (10μg/ml)	This study
pLC21	ΔBBaJ23100-cueO	pBR322	cm^r (10μg/ml)	This study
pLC22	ΔBBaJ23100-copA	pBR322	cm^r (10μg/ml)	This study
pLC23	ΔBBaJ23109-cusS	pBR322	cm^r (10μg/ml)	This study
pLC25	BBaJ23100-cueO $^{M440Q, M441Q}$	pBR322	cm^r (10μg/ml)	This study
pLC26	BBaJ23100-cueO$^{D439A, M440Q, M441Q}$	pBR322	cm^r (10μg/ml)	This study
pCWCu1	cusR-P_{cusR}-P_{cusC}-repL-sfGFP	pSC101	amp^r (50μg/ml)	2
pCWCu6	P_{copA}-repL-sfGFP	p15A	kan^r (50μg/ml)	This study
pCWCu31	P_{cusR}-P_{cusC}-repL-sfGFP	pSC101	amp^r (50μg/ml)	2

Table S2: Strains information.

Name	Host strain	Plasmid	Antibiotic	Source
DH5α	/	/	/	Laboratory
Cu1	DH5α	pCWCu1	amp^r	Laboratory
Cu6	DH5α	pCWCu6	kan^r	This study
Cu7	DH5α::ΔcopA	pCWCu6	kan^r	This study
Cu27	DH5α	pCWCu1/pLC8	amp^r+cm^r	Laboratory
Cu31	DH5α	pCWCu31	amp^r	Laboratory
Cu33	DH5α	pCWCu1/pLC17	amp^r+cm^r	This study
Cu38	DH5α::ΔcueO	pCWCu31	amp^r	This study
Cu39	DH5α::ΔcusCFBA	pCWCu31	amp^r	This study
Cu40	DH5α::ΔcueO ΔcusCFBA	pCWCu31	amp^r	This study
Cu68	DH5α::ΔcopA	pCWCu31	amp^r	This study
Cu69	DH5α	pCWCu31/pLC19	amp^r+cm^r	This study
Cu85	DH5α::ΔcusCFBA	pCWCu31/pLC20	amp^r+cm^r	This study
Cu90	DH5α::ΔcusCFBA	pCWCu31/pLC21	amp^r+cm^r	This study
Cu91	DH5α::ΔcusCFBA	pCWCu31/pLC22	amp^r+cm^r	This study
Cu92	DH5α::ΔcopA	pCWCu31/pLC22	amp^r+cm^r	This study
Cu93	DH5α	pCWCu1/pLC23	amp^r+cm^r	This study
Cu95	DH5α::ΔcusCFBA	pCWCu31/pLC25	amp^r+cm^r	This study
Cu96	DH5α::ΔcusCFBA	pCWCu31/pLC26	amp^r+cm^r	This study

Table S3: Primers information.

Name	Primer information	
F1	cg-GAATTC-atgctggctaaagtcact	Construction of pCWCu1
R1	act-GTCGAC-cagaaatcatccttagcgaaag	
F2	cg-GAATTC-ataatttctggtgattttatgccgccaactttactcg	
R2	acg-GTCGAC-ttactgaccatccggcacct	
F3	act-GTCGAC-tcatatttcctccgcatgttgc	Construction of pCWCu31
R3	act-GTCGAC-cagaaatcatccttagcgaaag	
Hg-f	ccacctgacgtctaagaaaccatt	Test for above plasmids
Hg-r	agcctgcataacgcgaagta	
pXW109As-SphI-f	acc-GCATGC-ctgggattacacatggcat	Construction of pCWCu6
pXW109As- SalI-r	atc-GTCGAC-ccagaaatcatccttagcg	
PcopAp- SalI-f	atc-GTCGAC-acccgcctggtttattaatttc	
PcopAp-SphI-r	tcc-GCATGC-gagtagcagcctgagtcgtta	
LJJ-pMT018/019-test-R1	cctttgagtgagctgataccg	Test for above plasmids
pMT027-test-f-0220	gcaactttatccgcctccatc	
plc8-hindIII-f	tgt-AAGCTT-ctcaggctgctactcaaaact	Construction of pLC8
plc8-xbaI-r	tgc-TCTAGA-tactttcctgtgtgactctagt	
cuss-xbaI-f	tgc-TCTAGA-atggtcagtaagccatttcagc	
cuss-hindIII-r2	tgg-AAGCTT-aggattaagcgggtaatgtgat	
plc9-VF	ctcaggctgctactcaaaact	Construction of pLC9
plc9-VR	aggattaagcgggtaatgtgat	
copa-F	cattacccgcttaatcct-aggaggaaaatat-atgtcacaaactatcgacctg	
copa-R	ttgagtagcagcctgag-cgcaatgatgtacttattccttcg	
plc17-vf	gttGacGgctagctcagtcctaggTacA-gtgctagctactagagtcacac	Construction of pLC17
plc17-vr	agcacTgtAcctaggactgagctagcCgtC-aacATCGATgaggtgaagac	
pLC4-f	acggttcctggccttttgctg	Test for above plasmids
pLC4-r	gaacctcttacgtgcccgat	
plc19f2	aga-GGGCCC-atgtcacaaactatcgacctgac	Construction of pLC19
plc19r2	agt-GGGCCC-tactttcctgtgtgactctagt	
plc20vf	act-GGGCCC-tacatcattgcgctcaggct	Construction of pLC20
plc20vr	cgg-GGTACC-tactttcctgtgtgactctagt	
cueO-if	tgg-GGTACC-atgcaacgtcgtgatttctt	
cueO-ir	tat-GGGCCC-cgtagcgcatcaggcagat	
pLC4-f	acggttcctggccttttgctg	Test for above plasmids
pLC4-r	gaacctcttacgtgcccgat	
pmt010-kana-f	taaatcggccatatcttcggcttcagactcgctgagcgcgtaactgtttg-ctcataggtgtcctggtaag	Knockout cueO
pmt010-kana-r	gcttatgcgctgccggatgatgtgaagtaagacttgctcagattgctgac-gcctcgtgatacgcctatt	

pTKRED-smr-f	gagcctatgtctccttgtaaacttctgccattttgtgtggcccttgcgct-taggcgcaatcactttcgtc	Knockout cusCFBA
pTKRED-smr-r	gtaatccgcgatgaagcgtcggctcttgattcgacgcagtatcttccgat-tccagaaccttgaccgaac	
test-cueo-f	cgcaacataatagctaacattgacc	Test
test-cueo-r	aggacagtatgccgttcccgat	
test-cusABFC-f1	gagcctggcgagtaaagttg	
test-cusABFC-r	caccagcacgaacattacccagt	
delcopA_pPB018_CmR-new-f	gtctaatcctgatgcaaatcgagccgattttttaatctttacggacttttt-taacgactcaggctgctact	Knockout copA and test
delcopA_pPB018_CmR-new-r	cgtcgatgccaaatgcgccaccctaaagcagcgcatccgcaatgatgtac-cgaaaactcacgttaagggatt	
test-copA-f1	ggaggtaattcctcaccccg	
test-copA-r1	tggctgaatatttggcacgc	
pLC21-f	cgg-AAGCTT-aaagaccgtattcggaaat	Construction of pLC21
pLC21-r	cgt-AAGCTT-catcgatgaggtgaagac	
pLC22-f	gct-AAGCTT-gtacatcattgcgctcagg	Construction of pLC22
pLC22-r	cgt-AAGCTT-catcgatgaggtgaagac	
pLC23-f	gat-GGGCCC-tcctaggaggaaaatatatg	Construction of pLC23
pLC23-r	taa-GGGCCC-catcgatgaggtgaagac	
pLC21-F	AGAAAGGCGGACAGGTATC	Test for above plasmids
pLC25-f	CGTGGGCGACcagcaaCTGCATCCGTTCCATATCCAC	Construction of pLC25
pLC25-r	ACGGATGCAGttgctgGTCGCCCACGCCAGAGATAAC	
pLC26-f	CGTGGGCGctcagcaaCTGCATCCGTTCCATATCCAC	Construction of pLC26
pLC26-r	ACGGATGCAGttgctgagCGCCCACGCCAGAGATAAC	
pLC25-F	GCATAACGCGAAGTAATC	Test for above plasmids
pLC25-R	GCTAAGGATGATTTCTGG	

REFERENCES

1. Wan, X., Volpetti, F., Petrova, E., French, C., Maerkl, S.J., and Wang, B. (2019). Cascaded amplifying circuits enable ultrasensitive cellular sensors for toxic metals. Nature Chemical Biology *15*, 540-548. 10.1038/s41589-019-0244-3.

2. Fu, Y., Li, J., Wang, J., Wang, E., and Fang, X. (2024). Development of a two component system based biosensor with high sensitivity for the detection of copper ions. Communications Biology *7*, 1407. 10.1038/s42003-024-07112-6.
